# Schizophrenia-Like Psychosis Presented in a Patient With a Temporal Lobe Tumor: A Case Report

**DOI:** 10.7759/cureus.29034

**Published:** 2022-09-11

**Authors:** Gerardo Romero-Luna, Sonia Iliana Mejía-Pérez, Jacqueline Ramírez-Cruz, Keren Magaly Aguilar-Hidalgo, Karla Marisol Ocampo-Díaz, Julia Moscardini-Martelli, Viviana Ramírez-Stubbe, José Omar Santellán-Hernández

**Affiliations:** 1 Radiosurgery, Instituto Nacional de Neurología y Neurocirugía "Manuel Velasco Suárez", Mexico City, MEX; 2 Neurosurgical Oncology, Instituto Nacional de Neurología y Neurocirugía "Manuel Velasco Suárez", Mexico City, MEX

**Keywords:** mass effect, meningioma, neurosurgery oncology, glioma, brain tumor, psychiatric effects

## Abstract

Psychiatric symptoms caused by brain lesions are not uncommon nowadays, caused by several different pathologies such as Alzheimer's, dementia, vascular and oncological diseases, etc. and they are known as neuropsychiatric or neurobehavioral symptoms, overlapping as mental health disorders. The most common primary brain tumors are gliomas, and the most common neuropsychiatric symptoms caused by them are depression, anxiety disorder, schizophrenia-like psychosis, anorexia nervosa, or cognitive dysfunction. We present a case of a 46-year-old male with no psychiatric familial history who started with a schizophrenia-like psychosis with hallucinations and, in consequence, killed his mother, symptoms which, after almost eight years, were known to be caused by a brain tumor.

## Introduction

Brain tumors and their treatments can cause several mood and behavioral or cognitive symptoms that occur or overlap, such as mental health disorders. These are called neuropsychiatric symptoms, also known as neurobehavioral symptoms [1].

In the United States, the annual incidence of primary brain tumors is nine per 100,000 and 8.3 per 100,000 for metastatic brain tumors. The most common primary brain tumors are gliomas that arise from glial tissue and account for 40-55% of all brain neoplastic lesions [2]. Many brain tumors present with specific neurologic symptoms and signs; they can be focal or due to mass effect, causing nausea, vomiting, and the most common symptom, headache. Psychiatric symptoms related to brain lesions that are presented as the only clinical manifestation of a brain tumor may mask the presence of a brain tumor due to the complete absence of neurological deficits, which may appear later when the tumor grows [2,3].

The incidence of psychiatric symptoms related to brain tumors ranges from 50%-78% [4] and approximately 80% of them have tumors in the frontal and limbic regions [5]. The most common psychiatric symptoms in patients with organic brain lesions are depression, anxiety disorder, schizophrenia-like psychosis, anorexia nervosa, or cognitive dysfunction [6]. The spontaneity of the presentation and the atypical and diffuse nature of the symptoms, together with the poor response to initial interventions, should guide the clinician towards a differential diagnosis beyond psychiatric pathologies [7].

This report presents the case of a 46-year-old male who debuted with a schizophrenia-like psychosis, hearing voices and incoherent thinking, committing murder a month later, and developing neurological symptoms. He was diagnosed with a brain tumor seen in a CT scan that was linked to neurological and psychiatric symptoms.

## Case presentation

We present the case of a 46-year-old male with an important family history of a father who died of an unspecified brain tumor, a mother deceased due to homicide, and a pathological personal history of mild head trauma due to a traffic accident in 2007.

According to an accompanying family member, the patient began the neuropsychiatric symptoms in 2012, starting with unspecified seizures and the onset of delusional ideas as he reported feeling like a god and seeing other people as animals that were his subjects. He continued to have similar outbreaks for several years without receiving medical attention. In September 2015, he was admitted to a psychiatric hospital due to a psychotic breakdown; however, only his physical symptoms and the acute episode were treated without delving into his medical history. In September 2020, he was hospitalized for the second time in the same hospital due to another psychotic break with hallucinations and repetitive thoughts. This time he was approached by the psychiatric service, which diagnosed him with schizophrenia and began medical treatment. He constantly presented with psychotic outbreaks, in which delusions and hallucinations predominated and became highly violent.

**Figure 1:**
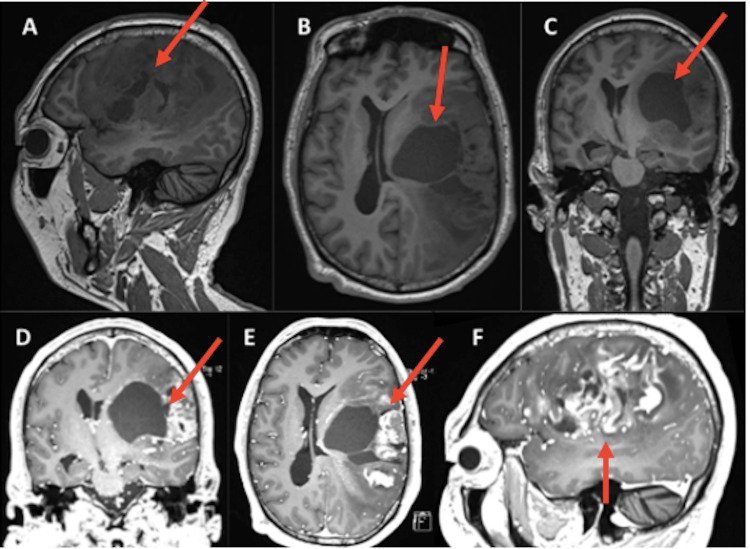
T1-weighted cranial MRI (A-C) and contrasted cranial MRI (D-F) A: sagittal section, diffuse tumor lesion in frontoparietotemporal region, heterogeneous, poorly defined borders, with central necrosis. B: axial section, tumor lesion with large cystic lesion corresponding to necrosis that displaces the midline structures, an insular component is observed. C, D: coronal section. E: axial section. F: sagittal section. Central necrosis is observed, with heterogeneous enhancement to the periphery.

In June 2021, the patient began to experience weakness in the right side of the body, decreased exteroceptive sensitivity, and motor aphasia, all of these neurological symptoms presented within a week. By November of 2021, he continued with symptoms in the motor sphere, undergoing a cranial MRI, which showed an intra-axial lesion in the mesial temporal area, orbital gyrus, and left to the insular area (Figure 1). By May 2022, the symptoms progressed causing headache, nausea, vomiting, atrophy and decreased exteroceptive sensitivity of the right side of the body, alterations in a language such as motor aphasia, difficulty in repetition, circumstantial speech with a tendency to tangentiality, right central facial paralysis, in addition to mystical religious delusions.

**Figure 2:**
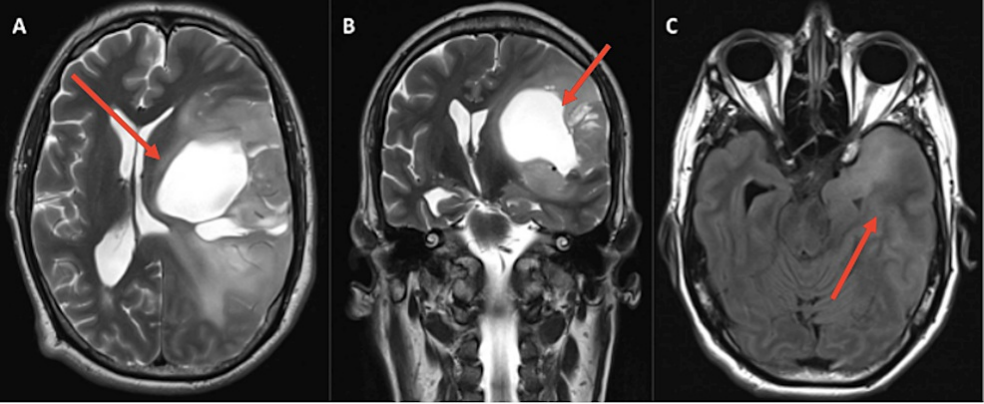
T2-weighted cranial MRI A (axial) and B (coronal): T2-weighted, cystic behavior, perilesional hyperintensity corresponding to tumor infiltration. Frontal-parietal-temporal lesion. C: Flair sequence, showing perilesional edema in the temporal pole and in the mesial temporal region.

**Figure 3:**
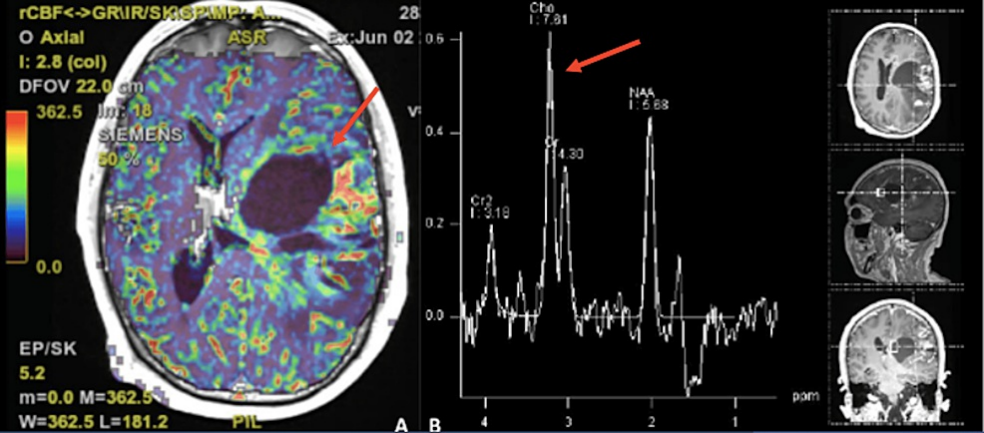
Cranial perfusion MRI (A) and spectroscopy (B) A: showing tumor periphery with hypervasculature. B: inversion of the curve with Choline peak in the central tumor area.

In July 2022, he presented conductive aphasia with right central facial palsy, right spinal nerve palsy, strength 0/5 and myotatic reflexes +++ in the right hemibody, spasticity, in the left hemibody he presented strength 4+/5 and reflexes ++; 40 points on the Karnofsky functional scale. He underwent an MRI showing a cystic behavior of the tumor (Figure 2) and the spectroscopy revealed a choline peak in the central portion of the tumor, referring to a high cellular division area (Figure 3). The patient is currently hospitalized in clinical custody because, presumably, during a psychotic outbreak, he attacked her mother and deprived her of life.

**Figure 4:**
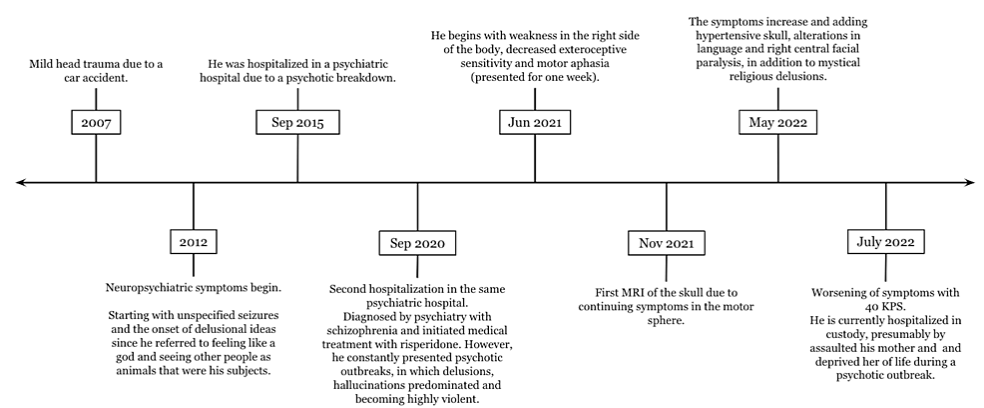
Timeline of the case presentation MRI - magnetic resonance imaging; KPS - Karnofsky Performance Scale

The subsequent treatment plan for the patient is based on palliative care since it was decided not to carry out any medical or surgical treatment for the extension of the tumor and its eloquent location in the brain (Figure 4).

## Discussion

A complete medical history and physical examination are mandatory to exclude psychiatric symptoms such as personality and mood changes, anxiety, anorexia, and atypical or new-onset symptoms that are refractory to treatment. It is necessary to rule out brain tumors by means of neuroimaging techniques, especially in patients presenting with atypical, new-onset, or treatment-resistant symptoms. Frontal lobe tumors are commonly associated with auditory and visual hallucinations, mania, panic attacks, or amnesia [8]. 

To illustrate, a frontal lobe tumor is likely to produce a "frontal lobe syndrome" that includes disinhibition, apathy, abulia, and deficits in executive function. A tumor of the dorsolateral prefrontal cortex is associated with problems in individual organization and planning; orbitofrontal tumors cause disinhibition, and mid-forehead tumors are associated with apathy and abulia [9].

The location of the tumor initially seemed to be the key to the association of neuropsychiatric symptoms; however, the literature has shown that there is no well-defined pattern for that since there are studies that report personality changes and emotional lability in tumors located outside the frontal lobe or in the limbic system as would be expected [10]. Interestingly, cerebellar tumors are also associated with aggressive behavior and personality changes [11]. In addition, posterior fossa and pineal meningiomas have been reported to present with peduncular hallucinosis, this refers to a psycho-sensory disorder consisting of multiple short-lived multiple-colored visual images occurring in the dark, and the patient is conscious that the images are not real [12].

In a 2015 meta-analysis by Madhusoodanan et al. of 148 cases of tumors with psychiatric symptoms, including 12 case series, 22% of cases had psychotic symptoms, and the most associated areas were the cerebral cortex, pituitary, pineal, and posterior fossa [15]. 

Younger patients may manifest these symptoms as anxiety, school phobia, psychomotor retardation, and mood swings. Patients with lesions of the ventral frontal cortex or lesions of the temporoparietal cortex reported significantly worse postoperative moods than patients with lesions elsewhere [13].

One in five patients with schizophrenia cared for in general psychiatric hospitals had an official criminal record. While most of these general psychiatric patients had committed non-violent crimes, 13% had committed violent crimes [14].

Schizophrenia and antisocial personality disorder (also alcoholism in one man) increased the likelihood of a homicide eight-fold and 10-fold, respectively. Madhusoodanan et al. showed that approximately 20% of psychotic patients who commit violent acts are motivated directly by delusions or hallucinations [15]. Psychiatric symptoms by brain region and tumor type are presented in Table 1. 

**Table 1:** Psychiatric symptoms by brain region and tumor type

Psychiatric symptoms	Authors	Tumor location	Tumor type
Psychosis (delusions/ hallucinations)	Kaloshi et al., 2013 [17]	Cerebellum	Extraventricular neurocytoma
	Canuet et al.,2011 [18]	Right parietal lobe	Meningioma
	Bunevicius et al., 2008 [4]	Left temporal lobe	Intra-cerebral cyst
		Left temporal lobe	Glioblastoma
	Rueda-Lara et al., 2003 [19]	Pituitary	Adenoma
	Craven, 2001 [20]	Pineal	Germinoma
	Filley et al., 1995 [21]	Temporal	Astrocytoma
	Madhusoodanan et al., 2007, 2015 [15,16]	Pituitary	Pituitary adenoma
		Cerebellum	Meningioma
		Pineal gland	Meningioma
		Temporal lobe	Oligoastrocytoma
		Left frontal lobe	Venous angioma
Mood (depression/mania)	Betul et al., 2011 [22]	Left temporal lobe	Glial tumor
	Cheema et al., 2010 [23]	Left frontal and temporal lobe	Glioblastoma
	Habermeyer et al., 2008 [24]	Right frontal lobe	Glioblastoma
	Bunevicius et al., 2008 [4]	Left temporal lobe	Intra-cerebral cyst
	Madhusoodanan et al., 2007, 2015 [15,16]	Left frontal lobe	Squamous cell carcinoma
		Left temporoparietal	Neurocytoma
		Thalamus	Unknown
	Madhusoodanan et al., 2007 [16]	Olfactory groove	Meningioma
		Right occipital, temporal and parietal	Metastatic lesions
		Right medial temporal	Astrocytoma
		Left posterior frontal	Glioma
Memory	Madhusoodanan et al, 2015 [15]	Thalamus	Unknown
		Corpus callosum	Unknown
		Temporal lobe	Unknown
		Frontal lobe	Unknown
Personality	Lajara-Nanson, 2000 [25]	Ventricular	Ventricular cyst
	Paul et al., 2000 [26]	Extramedullar with infiltration of the cerebral dura	Plasmocytoma
	Jones, 1993 [27]	Ventricular	Ventricular cysts
	Fulton et al., 1992 [28]	Frontal lobe	Multiple metastases
	Madhusoodanan et al., 2015 [15]	Right frontal lobe	Meningioma
		Temporal lobe	Unknown
Anorexia	Houy et al., 2007 [29]	Frontal side of the right Sylvian valley	Cavernous hemangioma
	Lin et al., 2003 [30]	Hypothalamic region	Unknown
	Climo, 1982 [31]	Hypothalamus	Craniopharyngioma
	Goldney, 1978 [32]	Hypothalamus	Craniopharyngioma
	White et al., 1977 [33]	Hypothalamus	Glioma
	Heron et al., 1976 [34]	Hypothalamus	Unknown
Anxiety	Madhusoodanan et al., 2015 [15]	Pituitary	Adenoma

In the situation of the patient in this clinical case, the patient's wife states that he began with a sudden change in personality; prior to this, he had not presented with behavioral alterations. Although the age of onset of symptoms would not be typical for a schizophrenia debut, without a previous study of the patient's personality, it is difficult to define whether the psychiatric illness began before the brain tumor or was caused by it. It is essential to highlight how complicated the association of tumors and their brain location with neuropsychiatric symptoms can be, even more so in those cases in which there is no improvement in the psychiatric disease after complete resection of the mass and in which there is no clear history of the patient's psychological condition. In addition, it is questioned to what extent the inflammation generated by the tumor can cause significant neurochemical changes, to the point of permanently manifesting a psychiatric disorder.

MRI is more efficient compared to CT scanning and is the procedure of choice when available. It is currently recommended that any patient older than 40 years with a change in mental cognitive, or emotional status, should have brain imaging, especially if there is no other clear alternative etiology. Brain imaging is recommended for all patients with early psychiatric symptoms, such as a psychotic break or personality change, starting at age 50 or if there is a change in the patient's symptoms or the patient develops suggestive neurological signs and symptoms [16]. In this case, the patient debuted with neuropsychiatric and neurological symptoms at the age of 36. Although there are several clinical reports and well-established indications to perform a neuroimaging study in these cases, no study was performed to exclude any structural pathology, delaying the proper diagnosis and changing the treatment possibilities. This highlights the importance of medical action guided by established algorithms to avoid malpractice.

The standard therapy to treat brain tumors is surgical resection followed by a combination of chemotherapy and radiotherapy. This may completely resolve the psychiatric symptoms, as most cases point to neoplasia as the underlying cause of the symptoms. Additionally, treatment of acute mass effects, such as increased intracranial pressure or hydrocephalus, can improve cognitive functioning and reduce psychiatric and behavioral symptoms. Excision of the neoplasm is usually followed by considerable improvement in psychiatric symptoms. However, in some cases, neuropsychiatric and behavioral symptoms persist or even worsen after surgical excision. In these circumstances, psychotherapeutic measures are instituted to improve the patient's clinical status and quality of life [16].

## Conclusions

Any patient with a sudden onset of psychiatric symptoms without a family or personal history of any psychiatric or psychological disorder should be searched for underlying pathology. The case presented in this report differs from others exposed in the literature due to the rapid progression of the symptoms developed and the action committed by the patient, leading him to commit the murder of a first-degree relative due to the psychiatric symptoms caused by the tumor.
